# Conventional versus Unconventional Oxygen Reduction Reaction Intermediates on Single Atom Catalysts

**DOI:** 10.1021/acsami.4c23082

**Published:** 2025-01-15

**Authors:** Tahereh Jangjooye Shaldehi, Soosan Rowshanzamir, Kai S. Exner, Francesc Viñes, Francesc Illas

**Affiliations:** 1 Hydrogen & Fuel Cell Research Laboratory, School of Chemical, Petroleum and Gas Engineering, 56944 Iran University of Science and Technology, Narmak, Tehran 16846−13114, Iran; 2 Faculty of Chemistry, Theoretical Catalysis and Electrochemistry, 27170 University Duisburg-Essen, Universitätsstraße 5, Essen 45141, Germany; 3 Cluster of Excellence RESOLV, Bochum 44801, Germany; 4 Center for Nanointegration (CENIDE) Duisburg-Essen, Duisburg 47057, Germany; 5 Departament de Ciència de Materials i Química Física & Institut de Química Teòrica i Computacional (IQTCUB), 16724 Universitat de Barcelona, c/Martí i Franquès 1-11, Barcelona 08028, Spain

**Keywords:** oxygen reduction reaction, unconventional mechanism, single atom catalyst, density functional theory, limiting potential, overpotential

## Abstract

The oxygen reduction reaction (ORR) stands as a pivotal process in electrochemistry, finding applications in various energy conversion technologies such as fuel cells, metal-air batteries, and chlor-alkali electrolyzers. Hereby, a comprehensive density functional theory (DFT) investigation is presented into the proposed conventional and unconventional ORR mechanisms using single-atom catalysts (SACs) supported on nitrogen-doped graphene (NG) as model systems. Several reaction intermediates have been identified that appear to be more stable than the ones postulated in the conventional mechanism, which follows the *OOH, *O, and *OH intermediates. This finding particularly holds for adsorbed *O_2_, which can have different adsorption geometries, ranging from η^1^Ο_2_ or η^2^Ο_2_ superoxo complexes as well as sin and anti complexes, with the two O-related ligands binding on the same or opposite sides, respectively. In the case of M@NG (M = Sc, Ti, V, Cr, Mn, Fe, Co, Ni, Cu, Zn, and Pt), the ORR follows these unconventional *O_2_ intermediates, whereas for Cr@NG and Cu@NG classical and unconventional *O_2_ intermediates compete. We approximate the electrocatalytic activity using the concept of the thermodynamic overpotential and demonstrate that the conventional mechanism gives rise to a smaller overpotential compared to mechanisms following unconventional intermediates during the four proton-coupled electron transfer steps. Our trend study indicates that transition metals with fewer *d* electrons reveal smaller electrocatalytic activity due to a larger thermodynamic overpotential. Among the investigated SAC systems, Co emerges as a promising candidate, with thermodynamic overpotential and limiting potential values of 0.38 and 0.85 V vs the standard hydrogen electrode, respectively, with the conventional mechanism being favored, and with Cu appearing as the second-best candidate.

## Graphical abstract

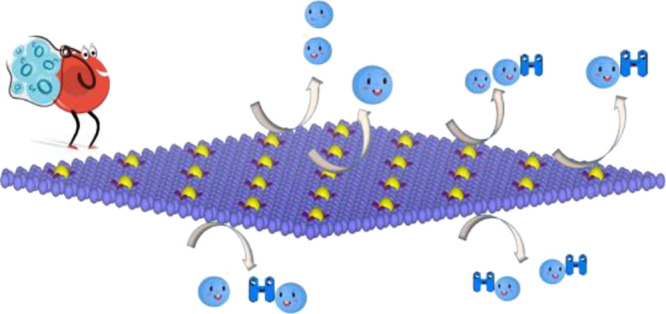

## 1 Introduction

The oxygen reduction reaction (ORR) holds a central position across various scientific, industrial, and environmental sectors due to its crucial involvement in processes spanning from energy conversion to environmental cleanup. In energy conversion, ORR plays a vital role in electrochemical devices like fuel cells, metal-air batteries, and chlor-alkali electrolyzers. For instance, fuel cells utilize ORR at the cathode to transform chemical energy into electrical energy, offering an appealing alternative to traditional power sources owing to their high efficiency and minimal environmental impact.^1,2^ Recent progress in catalyst design, including the creation of innovative materials such as carbon-based nanocomposites and transition metal oxides, has notably boosted ORR efficiency in fuel cell applications.^3–6^ In environmental remediation, the development of cost-effective ORR catalysts with superior catalytic activity and durability is crucial for the broad implementation of remediation technologies.^7^ Likewise, understanding ORR mechanisms in biological systems is imperative for comprehending the pathophysiology of diseases like cancer, neurodegenerative disorders, and cardiovascular diseases.^8^ Advancements in materials science and nanotechnology have facilitated the creation of novel ORR catalysts with tailored properties and improved performance. Nanomaterials like graphene, carbon nanotubes, and metal nanoparticles possess distinctive catalytic properties due to their large surface area and adjustable electronic structure, rendering them promising candidates for ORR applications.^9,10^ Furthermore, integrating ORR catalysts into nanocomposite structures enables the design of multifunctional materials with synergistic properties, thus opening new avenues for catalysis, sensing, and energy storage.^11,12^

Despite significant progress in ORR research, several challenges persist in fully realizing its potential across diverse applications. These challenges include developing stable and cost-effective catalysts, optimizing reaction kinetics and selectivity, and scaling up manufacturing processes.^13^ The kinetics of ORR can be sluggish, especially in acidic environments, resulting in reduced reaction rates and efficiency in electrochemical devices like fuel cells. Additionally, achieving high selectivity for the desired product, either H_2_O or H_2_O_2_ in fuel cells or for environmental applications, respectively, while minimizing side reactions remains a hurdle, affecting the overall performance and durability of ORR catalysts.^14–17^ Moreover, many ORR catalysts degrade over time due to chemical and electrochemical processes. Ensuring catalyst stability is crucial for prolonged device operation and can be compromised by factors such as dissolution, surface restructuring, and poisoning.^18^ The exorbitant cost of precious metal catalysts, notably platinum, restricts the widespread adoption of ORR-based technologies. Developing cost-effective alternatives with comparable performance poses a significant challenge. Furthermore, scaling up manufacturing processes to meet commercial demand without sacrificing catalyst quality and performance presents additional obstacles.^19,20^

The ORR mechanisms can vary significantly between acidic and alkaline electrolytes, complicating catalyst design and optimization. Creating catalysts that demonstrate high activity and stability under both acidic and alkaline conditions is essential for versatile applications in diverse electrochemical devices.^21,22^ Integration with other electrocatalytic processes is crucial in certain electrochemical applications, such as fuel cells and metal-air batteries, where ORR is often encountered as the limiting half-cell reaction.^23,24^ Addressing these challenges requires a multidisciplinary approach involving fundamental studies on reaction mechanisms, advanced materials synthesis and characterization techniques, computational modeling, and system-level engineering optimization. Collaborative efforts among academia, industry, and government institutions are imperative to overcome these hurdles and fully realize the potential of ORR in various electrochemical applications.^25–27^ Future research endeavors should concentrate on exploring novel materials and fabrication techniques, elucidating fundamental reaction mechanisms, and integrating ORR technologies into practical devices and systems.

The theoretical approach to the ORR primarily follows the four concerted proton–electron transfer (CPET) steps mechanism proposed by No̷rskov et al.^21^ where adsorbed OOH, O, and OH—commonly denoted as *OOH, *O, and *OH—appear as the reaction intermediates (Figure 1a–d). This is hereafter referred to as the conventional mechanism and has been broadly used, especially when the electrodes imply extended surfaces.^28^ However, in the case of single-atom catalysts (SACs), a comparison to prototypal systems in organometallic chemistry, suggests that other intermediates may also play a role. Inspired by this idea, Pacchioni et al.^29,30^ explored the mechanism of the oxygen evolution reaction (OER) for a collection of 30 SACs composed of 10 different metal atoms (Sc, Ti, V, Cr, Mn, Fe, Co, Ni, Pd, and Pt) anchored onto three commonly utilized 2D carbon-based materials, namely, graphene, nitrogen-doped graphene, and carbon nitride. Interestingly, none of the examined cases revealed the conventional pathway as the most favorable for the OER. Instead, in all instances, alternative intermediates exhibiting higher stability were reported as described in the next section.

**Figure 1:**
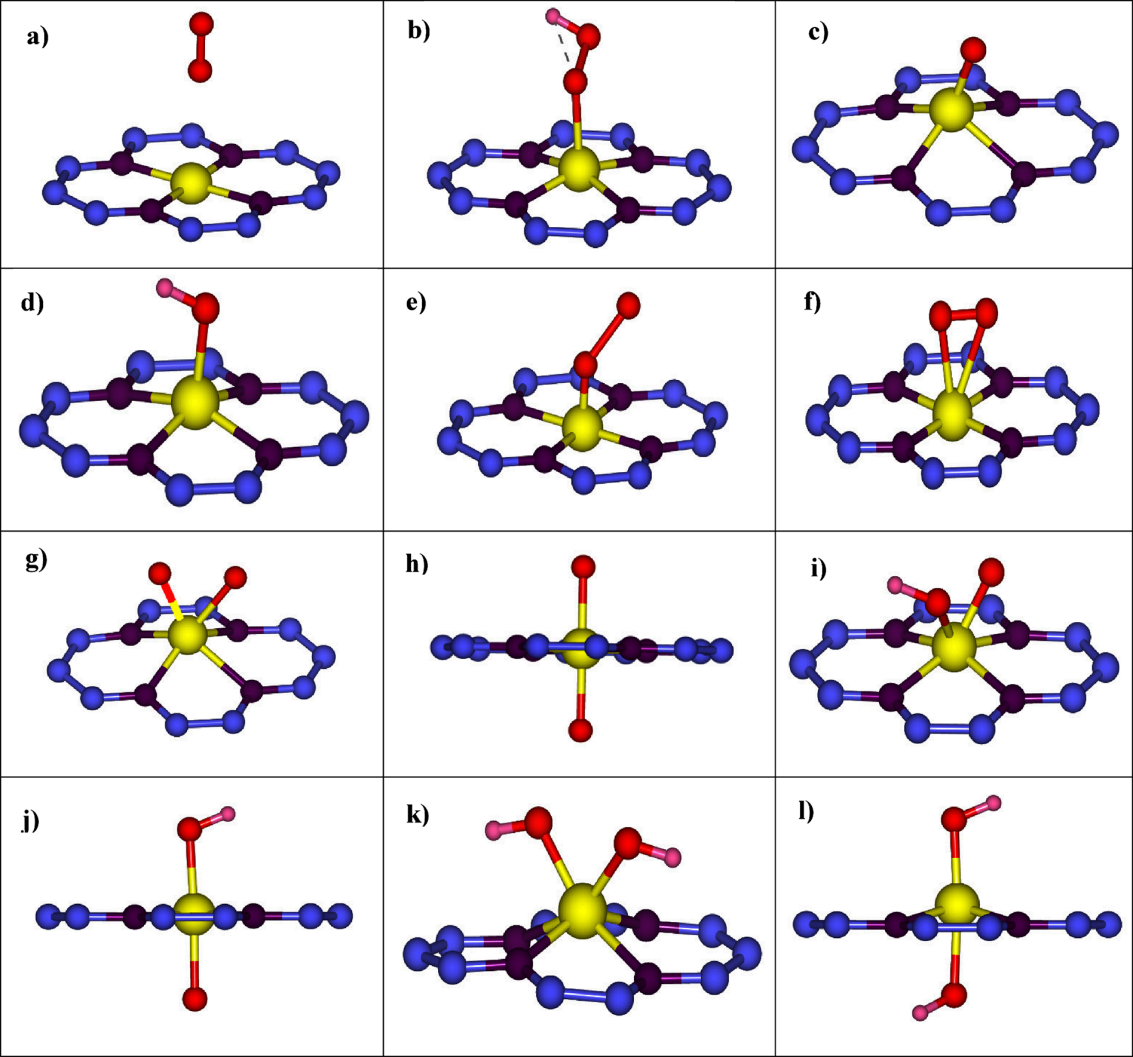
Possible ORR intermediates on SACs incorporated into N-doped graphene. These are physisorbed *O_2_ (a), *OOH (b), *O (c), *OH (d), η^1^ *O_2_ (e), η^2^ *O_2_ (f), sin-*O*O (g), anti-*O*O (h), sin-*O*OH (i), anti-*O*OH (j), sin-*OH*OH (k), and anti-*OH*OH (l). Blue, purple and yellow balls denote C, N, and the metal atom, respectively. Red and pink balls denote O and H atoms, respectively.

To shed light into this important issue related to the preferred ORR mechanism in acid media when using SACs, the present study investigates the conventional^21^ and unconventional^29^ ORR pathways for a single atom from the complete 3*d* transition series (Sc, Ti, V, Cr, Mn, Fe, Co, Ni, Cu, and Zn) supported on nitrogen-doped graphene with platinum (Pt) included for comparison. In line with the work of Barlocco et al.^29^ for SACs in the OER as well as general considerations by Exner on OER intermediates,^31,32^ our findings reveal deviations from the conventional ORR mechanism, with several SACs exhibiting unconventional behavior in certain intermediate species. Specifically, we identify stable intermediates that challenge traditional assumptions, shedding light on the underlying elementary steps and thermodynamics of ORR.

## 2 Conventional and Unconventional ORR Mechanism

Recent research endeavors have concentrated on unraveling the intricate details of ORR mechanisms in both acidic and alkaline media using advanced experimental and computational techniques.^33,34^ In this section, we briefly review the main features of the conventional and unconventional mechanisms.

### 2.1 Conventional ORR Mechanism

In acidic media, the ORR conventional mechanism^21^ can be summarized as in eqs 1–4:

O2(g)+*+H++e−→OOH* 1

OOH*+H++e−→O*+H2O(l) 2

O*+H++e−→OH* 3

OH*+H++e−→H2O(l)+* 4

where the chemical step O_2(g)_ + * → *O_2_ is usually not considered as it does not involve any CPET step. However, alternative pathways, such as the two-electron transfer mechanism leading to the formation of hydrogen peroxide (H_2_O_2_) as an intermediate product, have also been explored.^35^ The sluggish kinetics of ORR in acidic media necessitates efficient catalysts to enhance reaction rates and overall efficiency.^36^ Using density functional theory (DFT)-based methods and appropriate models for the electrode of interest, the Gibbs free energy Δ*G*_*i*_ for reactions 1–4 can be estimated utilizing methodologies extensively detailed in existing literature.^28^ The calculated Δ*G*_*i*_ values offer an estimation of the equilibrium potential required for each step (*E*_*i*_^*o*^) to occur on a given substrate since $E_{i}^{o}=-\frac{\Delta G_{i}}{e}$ , where *e* represents the electron charge.^21^ It is to be noted that, regardless of the catalyst employed, the sum of Δ*G*_*i*_ values must equate to −4.92 eV, representing the equilibrium Gibbs free energy for the overall reaction at *U* = 0 V vs reversible hydrogen electrode (RHE). Hence, it is postulated that an ideal catalyst would exhibit Δ*G*_*i*_ = −1.23 eV for any *i* at a potential of *U* = 0 V vs RHE—or Δ*G*_*i*_ = 0 eV for any *i* at pH = 0 and *U* = 1.23 V vs RHE—where the maximum deviation from this value defines the theoretical limiting potential and the resulting thermodynamic overpotential (see below).

Notably, the conventional ORR mechanism in alkaline media exhibits distinct characteristics from acidic conditions. Here, the four-electron transfer pathway is typically favored, resulting in the direct reduction of oxygen to hydroxide ions without the accumulation of peroxide or superoxide intermediates. The alkaline environment facilitates faster reaction kinetics compared to acidic media, presenting potential advantages for electrochemical devices like alkaline fuel cells, metal-air batteries, and chlor-alkali electrolyzers.^37–39^ The proposed steps for the ORR in alkaline media are as follows:

O2+*+H2O+e−→OOH*+OH− 5

OOH*+e−→O*+OH− 6

O*+H2O+e−→OH*+OH− 7

OH*+e−→OH−+* 8

which involves the *OOH, *O, and *OH intermediates as in the mononuclear mechanism in acidic media.^21,40^ In a prior investigation, we conducted a thorough examination of the oxygen reduction reaction mechanism in alkaline media, specifically focusing on single atoms of nonprecious metals.^41^ At pH = 14, the equilibrium Δ*G*_ORR_ at *U* = 0 V versus the standard hydrogen electrode (SHE) is −1.60 eV—equivalent to four steps ideally with Δ*G*_i_ = −0.40 eV—in contrast to the situation at pH = 0 where Δ*G*_ORR_ = −4.92 eV with four steps with Δ*G*_i_ = −1.23 eV. Consequently, at pH = 14, the equilibrium potential versus SHE is *U* = 0.4 V, and under this circumstance, Δ*G*_ORR_ becomes zero, wherein Gibbs adsorption energies are shifted by +*n*·*e*·*U*, contingent upon the number of electrons corresponding to each step. Clearly, for an ideal catalyst, Δ*G*_ORR_ = 0 eV is attained at *U* = 0.4 V vs SHE and pH = 14. However, such an ideal catalyst has not been found so far due to scaling relations between the intermediate states in the ORR,^42,43^ thus requiring further efforts to overcome the limitations imposed by the scaling relations.

### 2.2 Unconventional ORR Mechanism

In the alternative mechanism, the formation of other complexes such as superoxo (η^1^ *O_2_) and peroxo (η^2^*O_2_) as in Figure 1e,f is also possible, introducing an additional nonelectrochemical step starting from the O_2_ molecule in the gas-phase.^29,30,44^ Besides, it is also possible that the O_2_(g) molecule dissociates into two *O adsorbates in a chemical step. Therefore, one must also consider the following possibilities:

O2(g)+*→η1O2* 9

O2(g)+*→η2O2* 10

O2(g)+*→O*O* 11

which, as mentioned, do not involve any CPET. Starting from the most stable adsorbed *O_2_ species as in eqs 9–11, the reaction can follow through the classical four CPET steps as in eqs 5–8 with the only difference that the first CPET starts from *O_2_ and not from O_2(g)_. However, the first CPET step can lead to other intermediates as indicated in eqs 12 and 13,

η1O2*+H++e−→O*OH* 12

η2O2*+H++e−→OOH* 13

and also eqs 12 and 13 as explained below. Superoxide species typically reveal a η^1^ nature, characterized by a slight elongation of the O–O bond length compared to that of free O_2_ (1.25 Å) (cf. Figure 1e). Conversely, peroxo complexes display a η^2^ coordination, featuring significantly larger O–O bond distances ranging from 1.35 to 1.45 Å (cf. Figure 1f).^29^ As an alternative, a competitive species where two separate *O atoms are adsorbed on the same metal site,^45^ here termed *O*O, can also form as in eq 11, which after a CPET step form *OH*O or the *OOH complexes, according to eqs 14 and 15 (see Figure 1i,j):

O*O*+H++e−→O*OH* 14

O*O*+H++e−→OOH* 15

The *O*OH species as in eqs 12 and 14, where both an O atom and an OH group are bound to the transition metal (TM) in Figure 1k, can undergo a second CPET to release an *OH*OH complex^46^ as in eq 16:

O*OH*+H++e−→OH*OH* 16

In a recent study, Zhong and Li^47^ have shown that the *OH*OH complex exhibits greater stability compared to the *O intermediate. Nevertheless, the presence of the atypical *OH*OH intermediate does not preclude the subsequent progression of the reaction along the conventional pathway. This is because the unconventional *OH*OH intermediate can be also derived from the *OOH species, as indicated by eq 17

OOH*+H++e−→OH*OH* 17

Prior to H_2_O formation as described in eq 4, the *OH*OH complex has the potential to acquire two proton and electron pairs, providing an alternative pathway to the classical *O species outlined in eq 3, ultimately resulting in the sequential release of two water molecules, as indicated by eqs 18 and 19

OH*OH*+H++e−→OH*+H2O 18

OH*+H++e−→*+H2O 19

The complexity increases when accounting for the possibility that two distinct conformers may exist for each of the OH* OH*, O* O*, and OH* O* complexes on a single-atom catalyst integrated within a 2D material. Specifically, the two O-related ligands can either bind on the same side (sin) or opposite sides (anti) of the plane containing the metal atom, as depicted in Figure 1.

## 3 Methods and Models

The DFT calculations reported in the present work were carried out using the Vienna *Ab Initio* Simulation Package (VASP),^48^ with spin polarization taken into account in all cases. The Perdew–Burke–Ernzerhof (PBE)^49^ was chosen to account for exchange, correlation and missing kinetic energy effects, and was augmented with the D3 term developed by Grimme^50^ to include dispersion effects. This choice is supported by the evidence that PBE accurately describes the bulk and surfaces of the three transition metal series, as well as its reliable treatment of closed-shell metallic systems.^51,52^ However, it is important to acknowledge the inherent limitations of current functionals, particularly when describing gas-phase thermochemistry,^53^ since both the reactants and products are gas-phase molecules, and also when describing magnetic systems.^54^ The PBE errors in the gas-phase calculations can be corrected following previous work,^55^ as outlined below. Furthermore, employing a consistent method across different systems allows us to capture the key trends, which is the primary objective of this study. Another possible source of error is the existence of localized d-electrons which GGA functionals tend to excessively delocalize. This can be avoided by making use of hybrid functionals including a fraction of nonlocal Fock exchange such as PBE0 ^56,57^ or HSE06,^58^ or through the addition of the somehow empirical onsite two-electron repulsion term *U* leading to the basis of the PBE+*U* approach.^59^ However, one must advert that the choice of the contribution of Fock exchange in hybrid functionals, and also the range separation parameter in HSE06, or the value for the *U* parameter in PBE+*U* remain open issues as discussed elsewhere.^60–63^ Here, it is worth mentioning recent work by Barlocco et al.^64^ in systems similar to those studied in the present work where, for some SACs with 3*d* metals with magnetic ground state, the Δ*G* values obtained with PBE and PBE+*U* exhibit some differences, indicating that individual values have to be taken with caution. Nevertheless, since the main goal of the present paper is to compare the ORR energy profile for the conventional and unconventional mechanisms, one can safely compare the results obtained using the PBE functional as the main interest is in the energy differences and not in the numerical values. This claim is supported by comparison of the ORR profile for the case of Fe obtained from PBE and PBE+U profiles discussed in the next section.

The valence electron density was represented using a plane-wave basis set with a kinetic energy cutoff of 415 eV, and the Projector Augmented Wave (PAW) method proposed by Bloch,^65^ as implemented by Kresse and Joubert,^66^ was employed to consider the influence of core electrons on the valence electron density. Numerical integrations in reciprocal space were performed using a 4 × 4 × 1 mesh of special **k**-points.^67^ Structural optimization was iterated until the maximum force on any atom in the supercell was below 0.01 eV Å^–1^, with the total energy convergence criterion set to 10^–5^ eV.

In the framework of the Computational Hydrogen Electrode (CHE) model and for an actual catalyst, the potential at which all reaction steps are thermoneutral or downhill in free energy is referred to as the thermodynamic limiting potential (*U*_L_)^28^ and can be defined as

$$U_{L}=-\frac{1}{e}[\max(\Delta G_{i})]$$ 20

where *i* stands for all electrochemical steps involved and each Δ*G*_*i*_ is computed at *U* = 0 V or, equivalently,

$$U_{L}=U_{0}-\frac{1}{e}[\max(\Delta G_{i})]$$ 21

where each Δ*G*_*i*_ is computed at *U* = *U*_0_ *.*

For each *i* step, Δ*G*_*i*_ at *U* = 0 V is computed as

$$\Delta G_{i}=\Delta E_{i}-T\cdot \Delta S_{i}$$ 22

whereas at a generic potential *U* = *U*_*x*_

$$\Delta G_{i}(U=U_{x})=\Delta G_{i}+n\cdot e\cdot U_{x}$$ 23

where Δ*E*_*i*_ is the total DFT energy difference for this step including the contribution of the zero-point energy, Δ*S*_*i*_ is the corresponding entropy change which for adsorbed species includes the vibrational contribution to the partition function only whereas for gas phase molecules, it is taken for thermodynamic tables; *T* stands for temperature, taken usually as 298.15 K; and *n* corresponds to the number of electrons involved.^28^ Given that we focus on the free-energy changes of elementary steps, *n* = 1 in the remainder of this manuscript. Within this theoretical framework, the thermodynamic overpotential (η_TD_) is defined as the difference between the thermodynamic equilibrium potential (*U*_0_) and the limiting potential (*U*_L_), which is

$$\eta_{\mathrm{TD}}=U_{0}-U_{L}$$ 24

In acidic and alkaline media, the equilibrium potential *U*_0_ is +1.23 V and +0.4 V vs. SHE, respectively. For the ideal catalyst, there should be no overpotential, meaning that all steps exhibit identical Δ*G*_*i*_ values, resulting in *U*_L_ aligning with *U*_0_.^68,69^

The model for the SAC consists of a transition metal atom at the 4-fold site of N-doped graphene, and is represented by a sufficiently large 5 × 5 × 1 supercell of the one-layer pristine graphene with a starting C–C bond length at 1.42 Å, typical of pristine graphene, and lattice parameters of *a* = *b* = 12.3 Å and *c* = 20 Å. The latter value was specifically chosen to prevent interaction between periodic replicas. From the pristine graphene model, the N-doped graphene model was generated by substituting four C atoms by four N atoms. Finally, the SAC model was constructed by introducing a metal atom (Sc, Ti, V, Cr, Mn, Fe, Co, Ni, Cu, Zn, or Pt) into the cavity surrounding the N atoms and the resulting structure fully optimized with the PBE-D3 functional. This arrangement results in the metal atom forming two five- and two six-membered rings, as depicted in Figure 2. The final unit cell comprises 44 C atoms, 4 N atoms, and a single metal atom. Note that in the remainder of this manuscript, we consider adsorbates on the metal SAC center only, but not on the carbon or nitrogen support.

**Figure 2:**
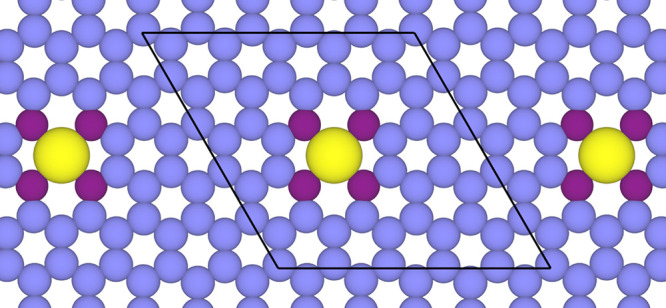
Schematic representation of the unit cell of the periodic model used to represent the SAC electrocatalysts used in the present work. These consist of a metal atom at the 4-fold site of N-doped graphene. Color code as in Figure 1.

Using the above-described model, the Gibbs free energy of each step was computed following each mechanism which also led to the predicted thermodynamic overpotential and limiting potential for each of the scrutinized SAC models. Finally, it is worth noting that, to account for the intrinsic errors of the approximate functionals,^55^ as the one used in the present work, the Gibbs free energy of the gas phase O_2_ molecule in its triplet electronic ground state, *G*(O_2_^(g)^), has been estimated from the experimental standard Gibbs free energy of liquid water phase formation (i.e., 4.92 eV), assuming that the error in the gas phase H_2_ and H_2_O molecules is negligible and accounting for a suitable liquid-phase correction for water.^70^ This implies that the PBE error for *G*(O_2(g)_) is 0.33 eV^70^ and, consequently, the *G*(O_2(g)_) value used in this work is −9.734 eV.

## 4 Results and Discussion

Before starting the discussion, we comment on details of the electronic ground state of the studied systems. For SACs with Sc, Ni and Zn, the ground state is nonmagnetic. For Sc and Ni, this is in agreement with previous work;^30^ for Zn this is not unexpected as this is not a magnetic element. For the rest of 3*d* transition metals the electronic ground state is magnetic with a magnetic moment, estimated from the total spin density in the unit cell, varying roughly between 1 and 3 as reported in Tables S1 and S2 in the Supporting Information (SI). For the SACs with magnetic ground state, the unpaired electron density is normally reduced when the different intermediates are adsorbed, as expected from the formation of additional covalent bonds with concomitant electron pairing. Finally, for the Pt@NG SAC, the electronic ground state is nonmagnetic, again in agreement with previous work.^30^

We start by analyzing the four-electron pathway for the ORR in acidic condition in the conventional mechanism as in eqs 1–4. For completeness, the calculated total energy, *E*, zero point energy contribution, *E*_ZPE_, temperature times entropy, *T*·*S*, and Gibbs free energy, *G*, values for *O_2_, *OOH, *O, and *OH on each metal are reported in Tables S3 to S14 of the Supporting Information, respectively. The steps with a positive Δ*G* value or slightly negative values are thermodynamically hindered and determine the theoretical overpotential. From the values in Table 1 and Figure S1 it is clear that for Sc, Ti, and Cr, there is only one step with positive Δ*G* that corresponds to *O to *OH conversion. For the V@NG system, there are two steps with positive Δ*G* but again with the *O to *OH conversion being the thermodynamically less favorable, thus defining the limiting potential. Thus, the calculated *U*_L_ vs SHE values for Sc, Ti, V, Cr, Mn, Fe, Co, Ni, Cu, Zn, and Pt are −2.08, −2.22, −1.56, −0.18, 0.36, 0.42, 0.85, 0.48, 0.52, 0.48, and 0.14 V. Since the difference between the equilibrium potential (*U* = 1.23 V vs SHE) and the limiting potential (*U*_L_) defines the thermodynamic overpotential η_TD_ (eq 24), one readily finds values of 3.31, 3.45, 2.79, 1.42, 0.87, 0.81, 0.38, 0.75, 0.71, 0.75, 0.75, and 1.09 V, in the same order, respectively. Based on these results, it is evident that transition metals with fewer *d* electrons exhibit a higher overpotential. Among the considered SAC systems, and assuming the conventional mechanism, Co emerges as the most promising candidate.

**Table 1:** Calculated Gibbs Free Energies (Δ*G*, in eV) for the Conventional Mechanism as in Equations 1–4, the Corresponding Overpotentials, and the Limiting Potential (η and *U*_L_, respectively, in V) for the Different Catalysts Explored as Computed at *U* = 0 V, pH = 0^a^

**SAC**	**Δ** *G*_**1**_	**Δ** *G*_**2**_	**Δ** *G*_**3**_	**Δ** *G*_**4**_	**η**_**TD**_	***U*_L_**
Sc	–3.75	–1.06	–2.19	2.08	3.31	–2.08
Ti	–6.13	–0.87	–0.14	2.22	3.45	–2.22
V	–5.91	–1.00	0.43	1.56	2.79	–1.56
Cr	–2.15	–2.85	–0.11	0.18	1.42	–0.18
Mn	–1.56	–2.44	–0.56	–0.36	0.87	0.36
Fe	–1.76	–1.98	–0.76	–0.42	0.81	0.42
**Co**	**1.24**	**1.21**	**1.63**	**0.85**	**0.38**	**0.85**
Ni	–0.48	–0.74	–1.91	–1.79	0.75	0.48
Cu	–0.52	–0.70	–2.07	–1.63	0.71	0.52
Zn	–1.29	–0.51	–2.63	–0.48	0.75	0.48
Pt	–0.14	–0.46	–2.2	–2.12	1.09	0.14

a The values for the case of the Co SAC are highlighted in bold as this corresponds to the most favorable case

Before describing the results for the unconventional mechanism, we comment on the relative stability of the different *O_2_ species at the metal site of the scrutinized SAC models as in the unconventional mechanism these play a key role. The stability is assessed from the calculated Δ*G* corresponding to eqs 9–11, which is summarized in Figure 3. The η^1^*O_2_ bonding mode appears to be the most stable for Sc and Cu whereas for some other metals, such as V, Co, Ni, and Pt, the most stable bonding mode is η^2^*O_2_. Noteworthily, in the case of Cr, Mn and Fe, the *O*O (anti) appears to be the most stable species whereas *O*O (sin) is the most stable one for Ti and Zn. These results already indicate that this adsorption step should be considered in ORR mechanism, in agreement with the ideas of Barlocco et al.^29^ for the oxygen evolution reaction.

**Figure 3:**
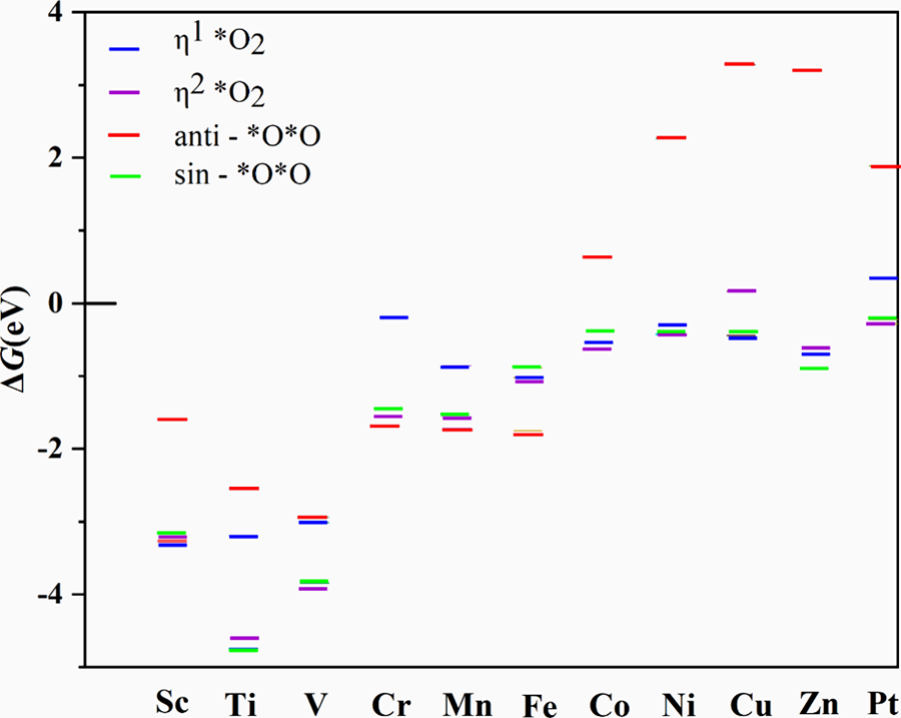
Relative stability of the different possible *O_2_ species at each one of the considered model catalyst.

For all the studied metals, our findings reveal stable unconventional intermediates in all ORR steps, and for Sc, Ti, V, Mn, Fe, Co, and Zn these are more stable than the conventional ones. In the case of Sc@NG, the η^1^*O_2_ superoxo complex, sin-*O*OH, sin-*OH*OH, and *OH from sin-*OH*OH species are more stable than their conventional counterparts and, hence, the ORR follows the unconventional mechanism. In a similar way, for Ti@NG and Zn@NG the sin-*O*O, sin-*O*OH, sin-*OH*OH and *OH from sin-*O*OH species are also more stable than those from the conventional mechanism. This also the case V@NG where the most favorable mechanism closely resembles the one predicted Ti@NG, except for the first step (η^2^*O_2_). In the case of Mn@NG, the anti-*O*O, sin-*O*OH, sin-*OH*OH, and *OH from sin-*OH*OH species are also more stable than those in the conventional mechanism. Finally, for Fe@NG, the anti-*O*O, anti-*O*OH, anti-*OH*OH, and *OH from sin-*OH*OH species are also more stable than those in the conventional mechanism. In the case of Ni and Pt, the η^2^*O_2_ superoxo complex, sin-*O*OH, anti-*OH*OH, and *OH from sin-*OH*OH species are more stable than their conventional counterparts, except the second step. This step is sin-*O*OH for Ni, and anti-*O*OH for Co and Pt, respectively.

For metals with large number of *d* electrons such as Cr and Cu, our findings reveal a competition between “conventional” and “unconventional” intermediates and, hence, with possibly a mixed mechanism. Interestingly, on this SAC, certain complexes exhibit markedly distinct stabilities. This is clearly seen in Figure 4, showing the ORR pathways where one intermediate conform to the classical type, while the other three are clearly unconventional. For instance, the sin-*OH*OH, and *OH from sin-*O*OH species and the η^1^*O_2_ superoxo complex are approximately 0.48 eV more stable than their classical counterparts for Cu. This introduces steps that significantly diminish the anticipated catalytic activity. For completeness, the calculated *E*, *E*_ZPE_, *T·S*, and *G* values for unconventional intermediates on each metal are reported in Tables S5 to S12 of the Supporting Information. From the values in Table 2 and Figure S2, it is clear that for Sc, Ti, Cr, Mn, Fe, and Pt, there is only one step with positive Δ*G* that corresponds to *O to *OH conversion. For the case of V, there are two steps with positive Δ*G* but again with the *O to *OH conversion being the thermodynamically less favorable, thus defining the limiting potential.

**Table 2:** Calculated Gibbs Free Energies (Δ*G*_*i’*_, in eV) for the Unconventional Mechanism with the Sequence of Steps Indicated in the Second Leftmost Column, the Corresponding Overpotentials, and the Limiting Potential (η and *U*_L_, Respectively, in V) for the Different Catalysts Explored as Computed at *U* = 0 V, pH = 0^a^

**SAC**	**sequence**	**Δ** *G*_1’_	**Δ** *G*_2’_	**Δ** *G*_3′_	**Δ** *G*_*4*’_	**Δ** *G*_5′_	**η**_**TD**_	***U*_L_**
Sc	9, 14, 16, 18, 19	–3.28	–0.67	–2.36	–0.83	2.22	3.45	–2.22
Ti	11, 14, 16, 18, 19	–4.75	–2.68	–1.13	–0.27	3.91	5.14	–3.91
V	10, 14, 16, 18, 19	–3.84	–2.89	0.05	–0.77	2.53	3.76	–2.53
Cr	11, 12, 6, 18, 19	–2.26	–1.27	–1.29	–0.47	0.37	1.60	–0.37
Mn	11, 14, 16, 18, 19	–1.72	–1.02	–1.26	–1.51	0.59	1.82	–0.59
Fe	11, 14, 17, 18, 19	–1.75	–0.85	–1.12	–2.22	1.02	2.25	–1.02
Co	10, 14, 16, 18, 19	–0.97	–0.45	–1.74	–1.03	–0.73	0.78	+0.45
Ni	10, 14, 17, 18, 19b	–0.43	–0.02	–2.13	–2.03	–0.31	1.21	+0.02
Cu	9, 1, 16, 18, 19	–0.44	–0.08	–1.76	–1.75	–0.89	1.15	+0.08
Zn	11, 12, 16, 18, 19	–0.65	–0.66	–1.06	–2.2	–0.32	0.91	+0.32
Pt	10, 14, 17, 18, 19	–0.27	–2.15	–2.08	–2.06	1.64	2.87	–1.64

a The prime added to the subindex is to distinguish from the CPET steps in the conventional mechanism. The values for the case of the Cu SAC are highlighted in bold as this corresponds to the most favorable case. Note also that the Δ*G*_1*’*_ corresponds to the formation of the *O_2_ species, which is not an electrochemical step.

**Figure 4:**
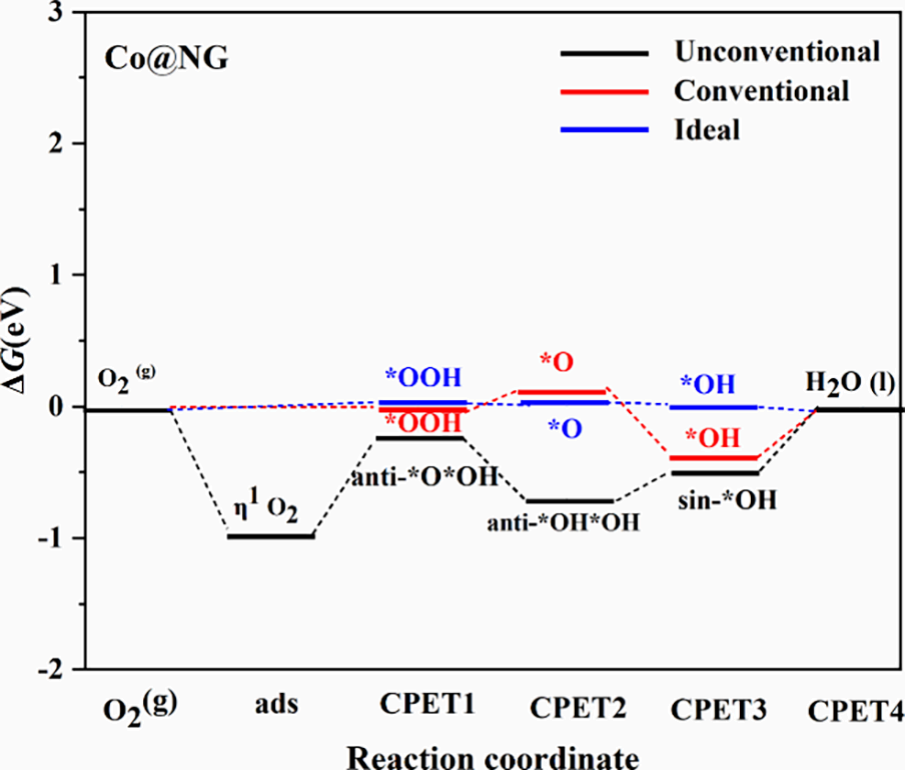
Gibbs free energy profile for the Co SAC at *U* = 1.23 V vs RHE, corresponding to the conventional calculated and ideal from eqs 1 to 4, and unconventional mechanisms with the sequence as in Table 2. The plot clearly shows that, in this case, the conventional mechanism is preferred.

Considering the ORR unconventional mechanism, the predicted U_L_ values for the Sc, Ti, V, Cr, Mn, Fe, Co, Ni, Cu, Zn, and Pt SACs are −2.22, −3.91, −2.53, −0.37, −0.59, −1.02, 0.45, 0.02, 0.08, 0.32, and −1.64 V vs SHE, respectively. According to eq 24, the difference between the equilibrium potential (*U* = 1.23 V vs SHE) and the limiting potential (*U*_L_) defines the thermodynamic overpotential η_TD_ with values of 3.45, 5.14, 3.76, 1.60, 1.82, 2.25, 0.78, 1.21, 1.15, 0.91, and 2.87 V vs SHE, in the same order, respectively. Among the studied SACs, Co emerges as the most promising candidate with lowest overpotential. Here, it is worth mentioning that, the overpotential values derived from the conventional mechanism tend to be lower compared to those obtained when accounting for unconventional ORR intermediates. Therefore, even if unconventional intermediates are found that need to be considered, their presence do not alter the predictions that arise from the conventional mechanism although it is also clear that they would cause a decrease of the catalytic performance.

To close this section, we briefly comment on the effect of the functional by taking Fe@NG as a case example which is justified by the fact that elements in the middle of the 3*d* transition metal period are those where spin polarization and dynamic electron correlation within the 3*d* shell are more important. Here we compare the *U*_L_ and η_TD_ values predicted by the PBE and the PBE+*U* functional with *U* = 4.5 eV which is a standard value. Note, also, that the choice of the *U* value is rather arbitrary, it has to be different for different transition metal elements and, except comparing to some well-defined experimental property such as e.g. the band gap in oxides, there is no way to properly determine the appropriate value. Therefore, the following results have to be taken with caution as they provide a rough estimate of the effect of the on-site 3*d* electron correlation on the quantities of interest. The PBE values for the *U*_L_ and η_TD_ are 0.42 and 0.81 V whereas at PBE+*U* level *U*_L_ increases to 0.63 V, and the overpotential decreases to 0.60 V. This 0.2 eV shift in the predicted *U*_L_ and η_TD_ underscores the influence of the functional choice in modifying the adsorption energies of intermediates, which directly affects the ORR mechanism and its energetics. Nevertheless, the qualitative Gibbs reaction profile remains unaltered and, more important, as the universal functional remains unknown, one has to rely on approximate functionals focusing on energy differences, likely to be less affected by the functional choice, rather than on the absolute values.

## 5 Conclusions

The present work evidence that the ORR mechanism on SACs at the N-doped graphene may deviate from conventional pathways, revealing more intricate trajectories than previously thought, which is in line with previous work on similar systems.^29,44^ Furthermore, we have identified several intermediates that are significantly more stable than the ones in the conventional mechanism. For Sc and Cu SACs the η^1^*O_2_ superoxo complex exhibits remarkable stability, but the η^2^*O_2_ superoxo complex is more stable for V, Co, Ni, and Pt. Interestingly, for Ti, Cr, Mn, Fe, Zn SACs, the *O*O intermediates (anti or sin) appear to the most stable. The case of Cu@NG, is especially interesting as one intermediate (*OOH) is as in to the conventional pathway, while three additional unconventional intermediates are found. Also, one intermediate (*O) is as in the conventional pathway, while three additional unconventional intermediates are found. For Sc@NG, Ti@NG, V@NG, Cr@NG, Mn@NG, Ni@NG, and Zn@NG, the sin-*O*OH is more stable than the *OOH intermediate, while the anti-*O*OH is preferred for Fe@NG, Co@NG, and Pt@NG. Moreover, the sin-*OH*OH is more stable for Sc@NG, Ti@NG, Mn@NG, Ni@NG, and Zn@NG while the anti-*OH*OH is preferred for Fe@NG, Co@NG, and Pt@NG. Transition metals with fewer *d* electrons exhibit a substantial limiting potential, which decreases markedly as the d shell approaches half-filled status. Among the SAC systems investigated, Co emerges as a particularly promising candidate, with a thermodynamic overpotential and limiting potential value of 0.38 and 0.85 V vs SHE, respectively, and with the conventional pathway being the thermodynamically preferred one. This prediction is supported by evidence that Co SACs show promising ORR activity and stability in alkaline/acidic media.^71–74^ While Co demonstrates superior catalytic activity and stability, Cu provides a quite good performance in specific metrics. The small but noticeable better performance of Co relative to Cu is offset by the significant cost benefits associated with Cu, as well as its wider availability. These advantages make Cu a highly attractive alternative for large-scale applications where cost-efficiency is paramount. Moreover, the promising activity of Cu-based catalysts suggests that further optimization could narrow the gap in performance, enhancing its potential as a sustainable and economically viable catalyst.

Finally, for ORR at this type of SACs, one must point out that, even if in some case the conventional pathway becomes the thermodynamically preferred one, the existence of stable unconventional intermediates may lead to notable decrease in the catalytic activity due to site blocking. Therefore, a systematic study of the possible unconventional intermediates appears as mandatory.

## Supplementary Material



## Notes

Calculated spin densities of the metal for both mechanism (Tables S1 and S2), total energy (*E*), zero point energy contribution (*E*_ZPE_), temperature times entropy (*T·S*), and Gibbs free energy (*G*) values for conventional and unconventional intermediates on each metal (Tables S2 to S6 and S7 to S14), Gibbs free energy diagrams for conventional and unconventional ORR mechanisms at *U* = 0 V and pH = 0 for the Sc, Ti, V, Mn, Fe, Ni, Cu, and Pt SACs with the ideal catalysts included for comparison (PDF)
