# Supplementary material for: Conventional versus Unconventional Oxygen Reduction Reaction Intermediates on Single Atom Catalysts
Source: ACS Appl Mater Interfaces. 2025 Jan 15;17(4):6450–9. doi: 10.1021/acsami.4c23082 (PMC12163882; doi:10.1021/acsami.4c23082)

Conventional *versus* Unconventional Oxygen Reduction Reaction Intermediates on Single Atom Catalysts

Tahereh Jangjooye Shaldehi,[1] Soosan Rowshanzamir,[1*] Kai S. Exner,[2] Francesc Viñes,[3] Francesc Illas[3*]

[1]*Hydrogen & Fuel Cell Research Laboratory, School of Chemical, Petroleum and Gas Engineering, Iran University of Science and Technology, Narmak, Tehran 16846–13114, Iran*

[2]*University Duisburg-Essen, Faculty of Chemistry, Theoretical Catalysis and Electrochemistry, Universitätsstraße 5, 45141 Essen, Germany; Cluster of Excellence RESOLV, Bochum, Germany; Center for Nanointegration (CENIDE) Duisburg-Essen, Duisburg, Germany.*

[3]*Departament de Ciència de Materials i Química Física & Institut de Química Teòrica i Computacional (IQTCUB), Universitat de Barcelona, c/ Martí i Franquès 1-11, 08028 Barcelona, Spain*

[*]Corresponding authors: francesc.illas@ub.edu, rowshanzamir@iust.ac.ir

Table S1. Calculated magnetic moments (μ_B in Bohr magnetons units), as estimated from the total spin density in the unit cell, assigned to the metal center, for the conventional mechanism of ORR on M@NG (M = Sc, Ti, V, Cr, Mn, Fe, Co, Ni, Cu, Zn, and Pt).

**Structure**	**Surface**	**$*O_2$**	***OOH**	***O**	***OH**
Sc	0	0.64	0	0.14	0
Ti	1.31	0	0	0	0.50
V	2.84	0.71	0	0.99	1.54
Cr	3.96	1.95	1.00	0.29	2.94
Mn	3.02	1.16	2.16	0.75	2.72
Fe	1.93	1.82	1.03	2.01	1.06
Co	0.69	1.05	0.00	1.64	0
Ni	0	1.89	0.84	3.3	0.67
Cu	0.99	0.79	1.46	1.81	1.14
Zn	0	1.43	0	1.07	0
Pt	0	1.88	0.88	0.25	0.83

Table S2. Calculated magnetic moments (μ_B in Bohr magnetons units), as estimated from the total spin density in the unit cell, assigned to the metal center, for the unconventional mechanism of ORR on M@NG (M = Sc, Ti, V, Cr, Mn, Fe, Co, Ni, Cu, Zn, and Pt).

**Structure**	**η^1 *O_2**	**η^2 *O_2**	**sin- *O*O**	**anti- *O*O**	**sin- *O*OH**	**anti- *O*OH**	**sin- *OH*OH**	**anti- *OH*OH**
Sc	0.64	0	0	0	0.82	1.34	0	0
Ti	0	0	0	1.32	0	0	0	0
V	0.98	0.71	0.71	0	0	0.74	0.73	1.19
Cr	1.92	1.91	1.91	0.98	0.99	0.71	1.74	2.50
Mn	0.02	2.59	2.59	0	2.32	0.42	3.10	2.62
Fe	1.82	1.83	1.07	0.73	3.43	1.81	1.65	1.41
Co	1.05	1.05	0.24	2.24	3.03	1.51	-2.27	-0.29
Ni	1.89	1.89	1.88	2.95	0.76	1.20	0.49	0.42
Cu	2.83	-1.0	2.81	4.28	0.60	3.12	0	2.03
Zn	0	0	1.46	1.61	0	1.61	0	0.56
Pt	0	1.88	1.88	0	0.87	0.85	0	0

Table S3. Calculated total energy (E), vibrational zero-point energy (E_{ZPE}), $T·S$, and Gibbs free energy for $*O_2$ (G_{*O2}) in ORR on M@NG (M = Sc, Ti, V, Cr, Mn, Fe, Co, Ni, Cu, Zn, and Pt) at $U = 0$ V.

Structure	E (eV)	E_{ZPE} (eV)	TS (eV)	G_{*O} (eV)
Sc	-460.63	0.75	0.35	-460.23
Ti	-462.29	0.80	0.26	-461.75
V	-461.98	0.79	0.25	-461.45
Cr	-460.80	0.76	0.39	-460.43
Mn	-459.71	0.79	0.28	-459.20
Fe	-458.52	0.79	0.29	-458.03
Co	-457.47	0.79	0.28	-456.96
Ni	-455.58	0.74	0.34	-455.17
Cu	-452.60	0.72	0.34	-452.23
Zn	-451.12	0.74	0.33	-450.70
Pt	-455.43	0.76	0.27	-454.94

Table S4. Calculated total energy (E), vibrational zero-point energy (E_{ZPE}), $T{\cdot}S$, and Gibbs free energy for *OOH (G_{*OOH}) in ORR on M@NG (M = Sc, Ti, V, Cr, Mn, Fe, Co, Ni, Cu, Zn, and Pt) at $U = 0$ V.

Structure	E (eV)	E_{ZPE} (eV)	TS (eV)	G_{*OOH} (eV)
Sc	-464.84	1.07	0.28	-464.05
Ti	-467.35	1.07	0.31	-466.59
V	-467.79	1.09	0.27	-466.97
Cr	-464.94	0.99	0.27	-464.21
Mn	-463.93	1.04	0.29	-463.18
Fe	-462.94	1.07	0.36	-462.23
Co	-461.45	1.10	0.31	-460.65
Ni	-459.32	1.06	0.42	-458.62
Cu	-456.38	1.04	0.41	-455.75
Zn	-455.34	1.03	0.38	-454.70
Pt	-458.93	1.06	0.33	-458.20

Table S5. Calculated total energy (E), vibrational zero-point energy (E_{ZPE}), TS, and Gibbs free energy for *O (G_{*O}) in ORR on M@NG (M = Sc, Ti, V, Cr, Mn, Fe, Co, Ni, Cu, Zn, and Pt) at U = 0 V.

Structure	E (eV)	E_{ZPE} (eV)	TS (eV)	G_{*O} (eV)
Sc	-454.79	0.69	0.24	-454.34
Ti	-457.19	0.72	0.21	-456.68
V	-457.71	0.72	0.20	-457.19
Cr	-456.82	0.73	0.19	-456.28
Mn	-455.38	0.72	0.19	-454.85
Fe	-453.95	0.71	0.19	-453.43
Co	-451.57	0.70	0.21	-451.08
Ni	-449.06	0.63	0.21	-448.64
Cu	-446.03	0.65	0.3	-445.68
Zn	-444.83	0.66	0.27	-444.43
Pt	-448.35	0.71	0.24	-447.88

Table S6. Calculated total energy (E), vibrational zero-point energy (E_{ZPE}), $T{\cdot}S$, and Gibbs free energy for *OH (G_{*OH}) in ORR on M@NG (M = Sc, Ti, V, Cr, Mn, Fe, Co, Ni, Cu, Zn, and Pt) at $U = 0$ V.

**Structure**	**E (eV)**	**E_{ZPE} (eV)**	**TS (eV)**	**G_{*OH} (eV)**
Sc	-460.66	0.96	0.28	-459.98
Ti	-460.95	0.93	0.26	-460.28
V	-460.90	0.95	0.26	-460.21
Cr	-460.57	0.94	0.22	-459.84
Mn	-459.60	0.95	0.21	-458.86
Fe	-458.41	1.00	0.23	-457.64
Co	-456.93	1.00	0.23	-456.16
Ni	-454.71	0.97	0.26	-454.00
Cu	-451.84	0.94	0.31	-451.21
Zn	-451.16	0.95	0.30	-450.5
Pt	-454.19	0.97	0.32	-453.54

Table S7. Calculated total energy (*E*), vibrational zero-point energy (E_{ZPE}), *T·S*, and Gibbs free energy for η^1*O_2 ($G_{\eta 1\ *O2}$) in ORR on M@NG (M = Sc, Ti, V, Cr, Mn, Fe, Co, Ni, Cu, Zn, and Pt) at *U* = 0 V.

Structure	*E* (eV)	E_{ZPE} (eV)	*TS* (eV)	$G\eta^1{}_{*O2}$ (eV)
Sc	-460.63	0.86	0.36	-460.13
Ti	-460.64	0.77	0.33	-460.2
V	-461.11	0.79	0.30	-460.62
Cr	-460.73	0.76	0.29	-460.26
Mn	-459.54	0.80	0.29	-459.03
Fe	-458.53	0.79	0.29	-458.03
Co	-457.38	0.79	0.32	-456.92
Ni	-455.58	0.75	0.33	-455.16
Cu	-452.58	0.71	0.37	-452.24
Zn	-450.62	0.74	0.41	-450.30
Pt	-454.68	0.89	0.45	-454.24

Table S8. Calculated total energy (E), vibrational zero-point energy (E_{ZPE}), TS, and Gibbs free energy for $\eta^2{*}O_2$ ($G_{\eta 2\ {*}O2}$) in ORR on M@NG (M = Sc, Ti, V, Cr, Mn, Fe, Co, Ni, Cu, Zn, and Pt) at $U = 0$ V.

Structure	E (eV)	E_{ZPE} (eV)	TS (eV)	$G\ \eta^2{}_{*O2}$ (eV)
Sc	-460.64	0.76	0.34	-460.22
Ti	-464.29	0.80	0.26	-463.75
V	-461.98	0.79	0.25	-461.44
Cr	-461.24	0.78	0.26	-460.72
Mn	-460.21	0.77	0.29	-459.73
Fe	-458.53	0.79	0.29	-458.03
Co	-457.38	0.79	0.33	-456.93
Ni	-455.58	0.74	0.34	-455.18
Cu	-451.93	0.73	0.42	-451.62
Zn	-450.62	0.74	0.38	-450.26
Pt	-455.42	0.76	0.22	-454.88

Table S9. Calculated total energy (E), vibrational zero-point energy (E_{ZPE}), $T·S$, and Gibbs free energy for sin-*O*O ($G_{sin\text{-}*O*O}$) in ORR on M@NG (M = Sc, Ti, V, Cr, Mn, Fe, Co, Ni, Cu, Zn, and Pt) at U = 0 V.

Structure	E (eV)	E_{ZPE} (eV)	TS (eV)	$G_{sin\text{-}*O*O}$ (eV)
Sc	-460.53	0.76	0.33	-460.1
Ti	-464.29	0.80	0.26	-463.75
V	-461.98	0.79	0.25	-461.44
Cr	-461.24	0.78	0.26	-460.72
Mn	-460.21	0.76	0.29	-459.74
Fe	-458.46	0.82	0.27	-457.91
Co	-456.64	0.78	0.27	-456.13
Ni	-455.60	0.75	0.31	-455.16
Cu	-452.61	0.72	0.33	-452.22
Zn	-451.07	0.74	0.26	-450.60
Pt	-455.38	0.76	0.21	-454.83

Table S10. Calculated total energy (E), vibrational zero-point energy (E_{ZPE}), $T{\cdot}S$, and Gibbs free energy for anti-*O*O ($G_{anti\text{-}*O*O}$) in ORR on M@NG (M = Sc, Ti, V, Cr, Mn, Fe, Co, Ni, Cu, Zn, and Pt) at $U = 0$ V.

Structure	E (eV)	E_{ZPE} (eV)	TS (eV)	$G_{anti\text{-}*O*O}$ (eV)
Sc	-458.94	0.78	0.30	-458.46
Ti	-460.04	0.78	0.29	-459.55
V	-461.10	0.81	0.26	-460.55
Cr	-461.46	0.81	0.22	-460.87
Mn	-460.50	0.84	0.23	-459.89
Fe	-458.47	0.82	1.13	-458.78
Co	-456.15	0.79	0.26	-455.62
Ni	-452.95	0.76	0.29	-452.48
Cu	-448.86	0.70	0.34	-448.5
Zn	-447.13	0.67	0.40	-446.86
Pt	-453.22	0.79	0.29	-452.72

Table S11. Calculated total energy (E), vibrational zero-point energy (E_{ZPE}), $T{\cdot}S$, and Gibbs free energy for sin-*O*OH (G_{*O*OH}) in ORR on M@NG (M = Sc, Ti, V, Cr, Mn, Fe, Co, Ni, Cu, Zn, and Pt) at U = 0 V.

Structure	E (eV)	E_{ZPE} (eV)	TS (eV)	$G_{sin\text{-}*O*OH}$ (eV)
Sc	-464.86	0.99	0.39	-464.26
Ti	-467.07	1.06	0.32	-466.33
V	-467.79	1.09	0.27	-466.97
Cr	-466.39	1.09	0.29	-465.59
Mn	-464.91	1.07	0.26	-464.1
Fe	-463.21	1.05	0.32	-462.48
Co	-460.85	1.03	0.37	-460.20
Ni	-459.33	1.07	0.38	-458.64
Cu	-455.55	0.72	0.33	-455.16
Zn	-455.37	1.03	0.37	-454.71
Pt	-458.94	1.06	0.34	-458.22

Table S12. Calculated total energy (E), vibrational zero-point energy (E_{ZPE}), $T{\cdot}S$, and Gibbs free energy for anti-*O*OH ($G_{anti\text{-}*O*OH}$) in ORR on M@NG (M = Sc, Ti, V, Cr, Mn, Fe, Co, Ni, Cu, Zn, and Pt) at U = 0 V.

Structure	E (eV)	E_{ZPE} (eV)	TS (eV)	$G_{anti\text{-}*O*OH}$ (eV)
Sc	-463.42	1.00	0.33	-462.75
Ti	-466.04	1.06	0.32	-465.30
V	-466.87	1.07	0.30	-466.10
Cr	-466.41	1.10	0.27	-465.59
Mn	-465.20	1.10	0.30	-464.40
Fe	-463.85	1.08	0.30	-463.07
Co	-461.62	1.07	0.30	-460.84
Ni	-458.53	1.05	0.32	-457.80
Cu	-454.85	1.28	0.40	-453.97
Zn	-453.59	0.96	0.43	-453.06
Pt	-459.09	1.06	0.30	-458.33

Table S13. Calculated total energy (*E*), vibrational zero-point energy (E_{ZPE}), $T{\cdot}S$, and Gibbs free energy for sin-*OH*OH ($G_{sin\text{-}*OH*OH}$) in ORR on M@NG (M = Sc, Ti, V, Cr, Mn, Fe, Co, Ni, Cu, Zn, and Pt) at $U = 0$ V.

Structure	*E* (eV)	E_{ZPE} (eV)	*TS* (eV)	$G_{sin\text{-}*OH*OH}$ (eV)
Sc	-470.87	1.26	0.45	-470.06
Ti	-471.96	1.35	0.31	-470.92
V	-471.43	1.36	0.29	-470.36
Cr	-470.46	1.37	0.28	-469.37
Mn	-469.27	1.31	0.31	-468.27
Fe	-467.32	1.34	0.34	-466.32
Co	-465.52	1.33	0.30	-464.49
Ni	-463.84	1.34	0.36	-462.86
Cu	-461.35	1.31	0.34	-460.38
Zn	-460.06	1.26	0.46	-459.25
Pt	-463.42	1.33	0.31	-462.40

Table S14. Calculated total energy (E), vibrational zero-point energy (E_{ZPE}), $T \cdot S$, and Gibbs free energy for anti-*OH*OH ($G_{anti\text{-}*OH*OH}$) in ORR on M@NG (M = Sc, Ti, V, Cr, Mn, Fe, Co, Ni, Cu, Zn, and Pt) at $U = 0$ V.

Structure	E (eV)	E_{ZPE} (eV)	TS (eV)	$G_{anti\text{-}*OH*OH}$ (eV)
Sc	-469.33	1.31	0.38	-468.4
Ti	-470.69	1.31	0.34	-469.72
V	-470.96	1.30	0.41	-470.07
Cr	-470.88	1.32	0.31	-469.88
Mn	-470.12	1.33	0.30	-469.09
Fe	-468.73	1.36	0.28	-467.65
Co	-467.09	1.37	0.32	-466.03
Ni	-464.41	1.36	0.33	-463.38
Cu	-460.82	1.29	0.40	-459.93
Zn	-459.48	1.22	0.55	-458.81
Pt	-464.86	1.38	0.38	-463.86

Figure S1. Gibbs free energy diagrams for conventional mechanism of ORR at $U = 0$ V and $pH = 0$ for the Sc, Ti, V, Cr, Mn, Fe, Co, Ni, Cu, Zn, and Pt SACs with the ideal catalysts included for comparison. The inset corresponds to the plots for the Co SAC at $pH = 0$ and $U = 0$ V, at the equilibrium potential (1.23 V) and at U_L *vs.* SHE, which is system dependent.

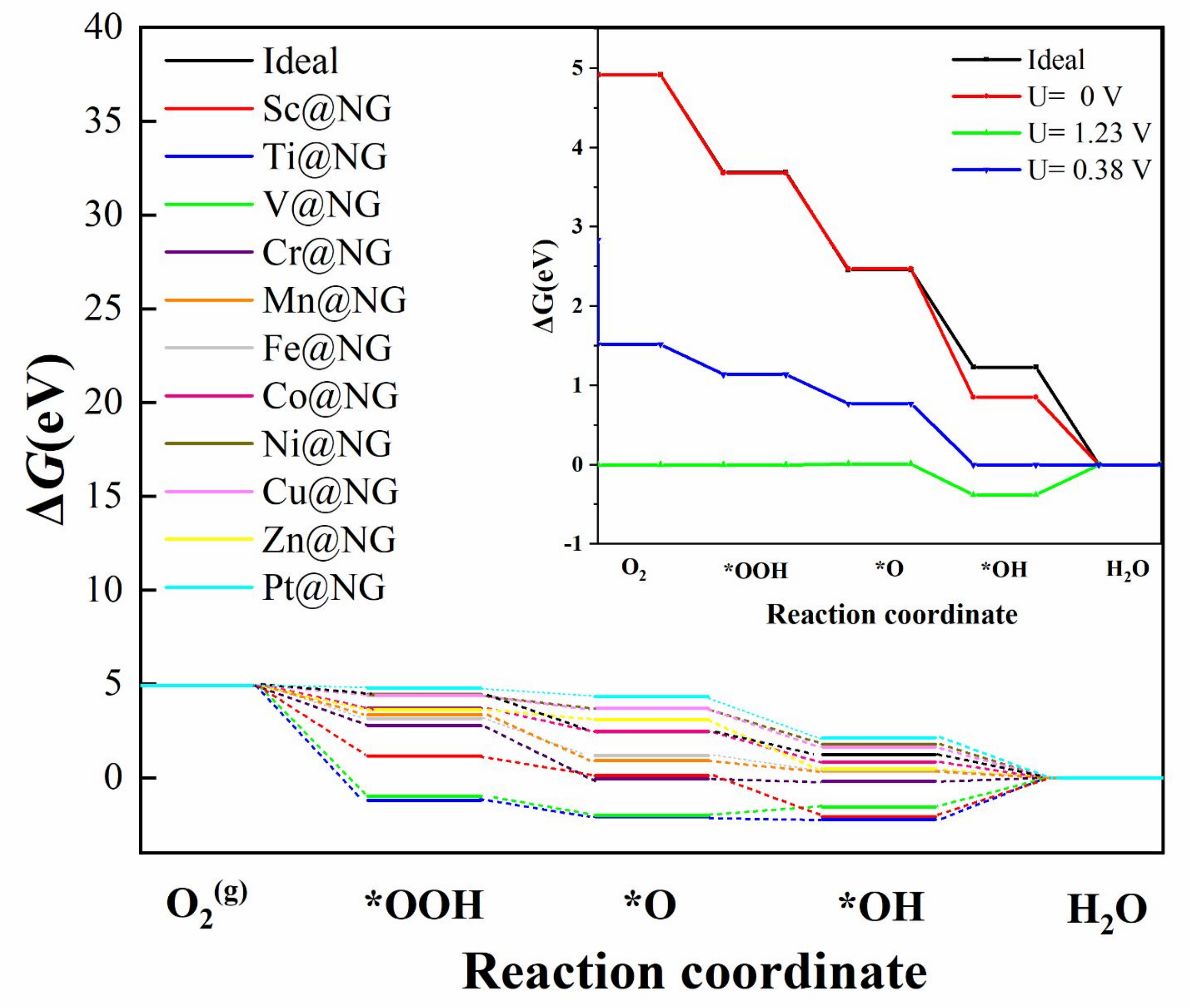

Figure S2. Gibbs free energy diagrams for unconventional mechanism of ORR at $U = 0$ V and $pH = 0$ for the Sc, Ti, V, Cr, Mn, Fe, Co, Ni, Cu, Zn, and Pt SACs with the ideal catalysts included for comparison. The inset corresponds to the plots for the Co SAC at $pH = 0$ and $U = 0$ V, at the equilibrium potential (1.23 V) and at U_L *vs.* SHE, which is system dependent.

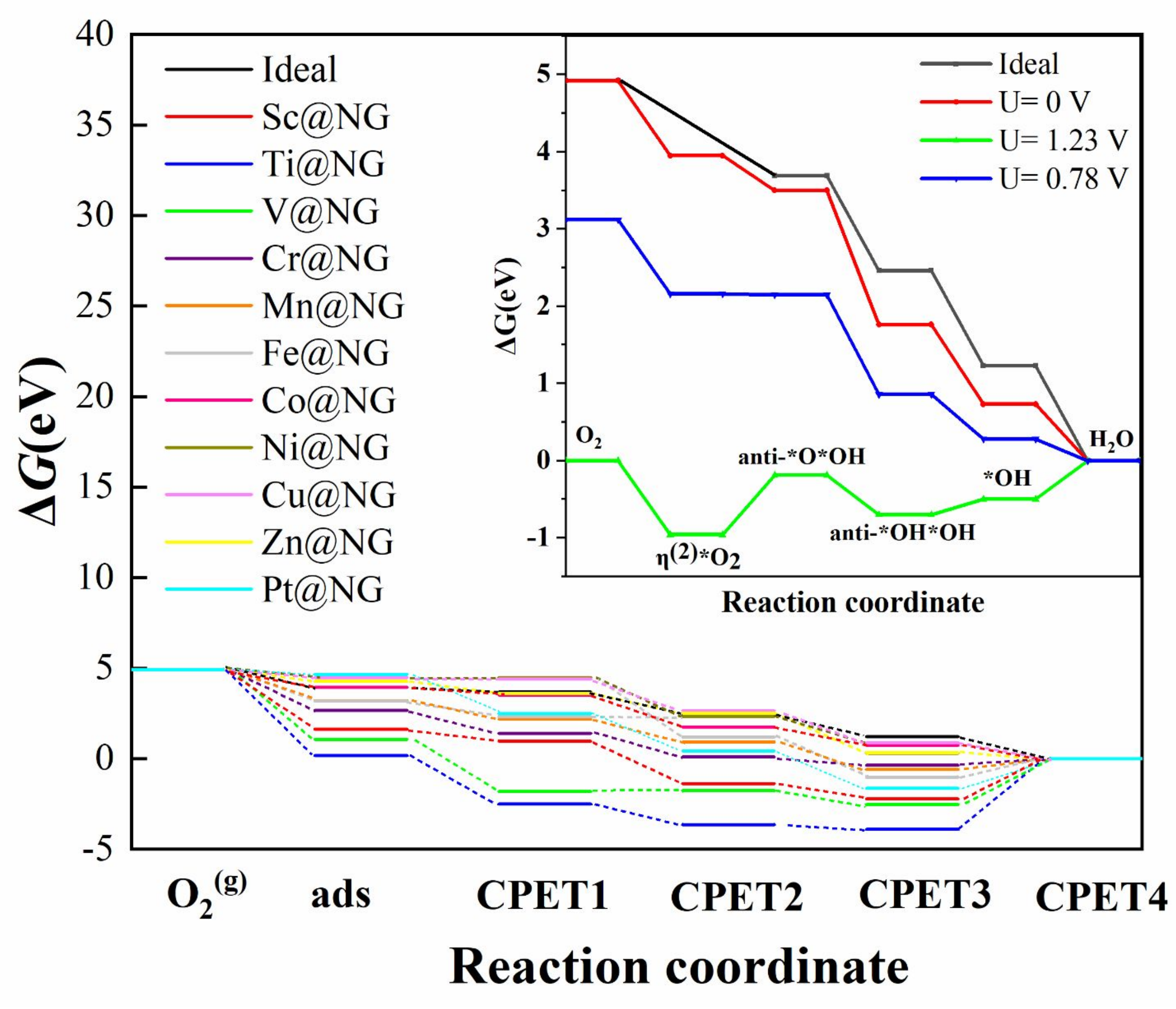

Supplement: Supplementary file 1 [file am4c23082_si_001.pdf]
